# Mesenchymal Stromal Cells: Heterogeneity and Therapeutical Applications

**DOI:** 10.3390/cells12162039

**Published:** 2023-08-10

**Authors:** Meryem Ouzin, Gesine Kogler

**Affiliations:** Institute for Transplantation Diagnostics and Cell Therapeutics, University Hospital Düsseldorf, 40225 Düsseldorf, Germany; gesine.koegler@med.uni-duesseldorf.de

**Keywords:** mesenchymal stromal cells, stem cells, heterogeneity

## Abstract

Mesenchymal stromal cells nowadays emerge as a major player in the field of regenerative medicine and translational research. They constitute, with their derived products, the most frequently used cell type in different therapies. However, their heterogeneity, including different subpopulations, the anatomic source of isolation, and high donor-to-donor variability, constitutes a major controversial issue that affects their use in clinical applications. Furthermore, the intrinsic and extrinsic molecular mechanisms underlying their self-renewal and fate specification are still not completely elucidated. This review dissects the different heterogeneity aspects of the tissue source associated with a distinct developmental origin that need to be considered when generating homogenous products before their usage for clinical applications.

## 1. Introduction

Mesenchymal cells were first discovered almost 60 years ago by Friedenstein et al. in the bone marrow of guinea pigs and were first described as in vitro colony-forming fibroblasts (CFU-Fs) [1]. These were characterized by their high replicative capacity and their ability to give rise to different cells of the non-hematopoietic lineage and to form osseous tissue in vivo. The term “mesenchymal” was adopted in the 1990s based on their multi-lineage differentiation capacities into mesodermal cell lineages both at population and clonal levels [2]. Maureen et al. suggested using the term “stromal stem cells” to distinguish them from histogenetically distinct hematopoietic and endothelial cells and to underline their capacity to maintain hematopoietic stem cells (HSCs) in the bone marrow [3]. The International Society for Cellular Therapy (ISCT) recommended the term “mesenchymal stromal cells” to avoid potential confusion, since the commonly used term “stem cell” should be reserved for the subset of cells possessing stem cell activity, designated by stringent and generally accepted criteria [4]. Bianco et al. elucidated in a large review the definition and functional identification of a mesenchymal stem cell-based on functional assays [5]. The main marker for the identification of a mesenchymal stromal cell was defined as the in vivo generation of heterotopic “ossicles” [5]. 

**Figure 1:**
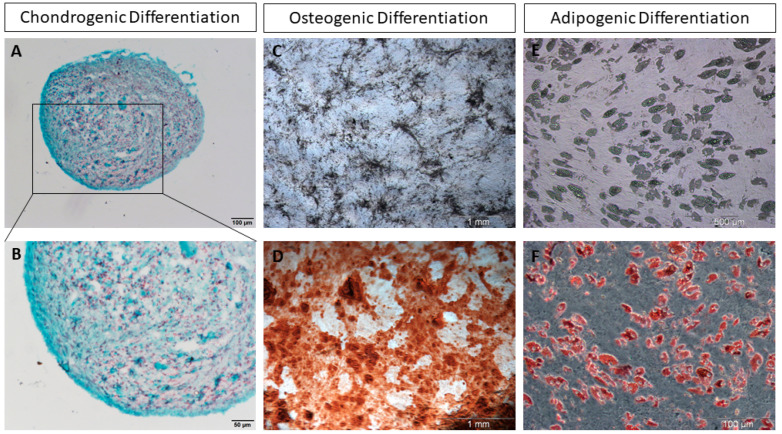
In vitro differentiation of hBM-MSCs. (**A**,**B**) Safranin O staining of a hBM-MSC chondrogenic pellet at day 21 of in vitro differentiation; (**C**) In vitro osteogenic differentiation of hBM-MSCs; (**D**) Alizarin Red S staining of hBM-MSCs at day 14 of in vitro osteogenic differentiation; (**E**) In vitro adipogenic differentiation of human hBM-MSCs; (**F**) Oil Red O staining of hBM-MSCs at day 21 of in vitro adipogenic differentiation. hBM-MSCs: Human bone marrow-derived mesenchymal stromal cells.

Due to their self-renewing capacity, their highly proliferative state, and their differentiation potential into cells of mesenchymal tissues including bone, fat, and cartilage, MSCs have gained growing attention in the last decade in the fields of tissue engineering and cell therapy (Figure 1). They became an attractive source in clinical applications for the regeneration of damaged tissues and the treatment of a broad range of human diseases [6]. 

Soon after their first isolation, MSCs became one of the most controversial areas in the field of stem cell biology. This is due to the complexity of their anatomical identity, heterogeneity, phenotype diversity, tissue distribution, lineage, and function. Nowadays, two different definitions of “MSCs” can be found in the literature. One that considers “MSCs” as cultured bone marrow stromal cells, which are progenitors specific to the bone marrow and not found elsewhere, characterized by their multipotency to exclusively form cells of the skeletal tissue and by their self-renewing capacity [5]. An important function of this progenitor cell is the maintenance and regulation of hematopoiesis, thus forming the hematopoietic stem cell niche in the bone marrow, which additionally gives structural support, facilitates migration, and regulates endocrine function [5,7]. The second definition considers “MSCs” as a range of progenitor cells that can differentiate into different lineages in vitro and reside beyond the bone marrow and the skeletal tissues [6,8,9].

MSCs can be harvested without major ethical concerns and have been shown to promote endogenous tissue repair and regeneration. This is largely related to their paracrine and immunosuppressive activities resulting in the alteration of the host immune response upon transplantation [10]. In consequence, various experiments and trials emerged showing the efficacy and effectiveness of MSCs as a promising alternative to conventional immune suppressants for the reduction of the progression of the graft-versus-host disease (GvHD), for example in the case of hematopoietic stem cell transplantation or in patients with severe treatment-resistant GvHD of the gut and liver [11]. 

Bone marrow-derived MSCs (BM-MSCs) were shown to modulate innate and adaptive immune responses [12]. Generally, several studies demonstrated the ability of MSCs to suppress T cell proliferation and pro-inflammatory cytokine secretion [13], dendritic cell maturation and their differentiation from monocytes through secretion of prostaglandin E2 (PGE2) and interleukin 6 (IL-6) [14,15]. Moreover, BM-MSCs were shown to interact with natural killer cell (NK cells) by inhibiting interleukin 2 (IL-2) induced proliferation of resting NK cells and partially inhibiting NK cell proliferation thus increasing their cytotoxicity [16]. Others reported that MSCs can interact with macrophages, thus increasing their adhesion to T cells and indoleamine 2,3-dioxygenase (IDO) expression and resulting in increased immunosuppressive capacities [17]. B cell proliferation can also be modulated by MSCs [18], which were shown to inhibit B cell terminal differentiation [19] and apoptosis [20]. However, the exact underlying mechanisms of action supporting the control of aberrant immunosuppressive responses remain to be elucidated. 

MSCs were also used for stem cell therapy of heart diseases such as myocardial infarction [21], pulmonary arterial hypertension [22] and coronary heart disease [23], as transplanted MSCs are able to engraft and differentiate into cells of the cardiac tissue e.g., cardiomyocytes and vascular cells. This is confirmed by an increased expression of the cardiac marker troponin T [24]. Moreover, they secrete paracrine factors that benefit cardiac repair by their immunomodulatory [25] and anti-fibrotic effects [26], but also through promotion of neovascularization [27].

In this comprehensive review, we aim to address several aspects of MSC heterogeneity, which forestalls their full exploitation in clinical application. Examples of current MSC advances and applications in clinical trials are presented.

## 2. Donor-to-Donor Heterogeneity

MSCs have been shown to display a high donor-to-donor biological heterogeneity, which should be taken into consideration for large-scale expansion. MSCs derived from 17 healthy bone marrow donors showed discrepancies in various aspects including osteogenic potential capacity, expression of alkaline phosphatase and growth rate [28]. These differences might additionally be enhanced by distinct factors including donor age, sampling bias during marrow aspiration and cell expansion conditions [29]. Donor-dependent heterogeneity is also related to the difficulty of the identification of MSCs, which is caused by the lack of unique and distinct cell features and the broad range of morphological properties. The subpopulations with distinct morphologies might also differ in their intrinsic properties. Given this heterogeneity within the same species, tissue, population and donor, which is partially responsible for the incongruence of the MSC-based clinical data, the ISCT additionally defined minimal criteria to characterize MSCs and minimize differences between laboratories worldwide. They were defined by their ability to adhere to plastic under standard culture conditions, by the expression of following surface markers: CD44, CD90, CD105, CD73 and by the lack of expression of the hematopoietic markers CD11b, CD14, CD19, CD34, CD45, CD79 and HLA-DR surface markers (Table 1) [9]. Furthermore, MSCs must possess the in vitro differentiation ability into chondrocytes, osteocytes and adipocytes [9]. 

Donor age is an important parameter that affects the functionality of MSCs, including their differentiation potential, self-renewal capacity, immunomodulatory properties, and tissue repair capacities if MSCs are harvested from the bone marrow. MSCs collected from older donors are characterized by a high amount of senescent and apoptotic cells, correlating with slow proliferation rates and population doubling times [9]. In addition, donor age negatively influences the ability of MSCs to form osteoblasts and weakens their repair capacity through the reduction of the immunomodulatory effects and the response to oxidative stress in comparison to cells harvested from younger donors. Kanawa et al. found that human BM-MSCs harvested from older donors showed a decreased chondrogenic potential along with a decreased expression of glycosaminoglycans (GAG), Sox9, collagen II, and aggrecan but did not affect the osteogenic or adipogenic potentials [30]. Other groups reported a decreased adipogenic and osteogenic potential of BM-MSCs with increasing donor age, with no changes in the chondrogenic differentiation potential [31,32].

Siegel et al. compared human BM-MSCs isolated from 53 different donors (25 female, 28 male; age: 13 to 80 years) and showed differences in phenotypes, with higher levels of CD71^+^, CD90^+^, CD106^+^, CD140b^+^, CD146^+^, CD166^+^, and CD274^+^ subpopulations in samples from younger donors [32]. These markers, however, did not correlate to donor age on the transcriptional level [32,33]. No correlation of donor age with the multi-lineage differentiation potential of the BM-MSCs could be confirmed [32].

Mareschi et al. isolated and expanded MSCs from the bone marrow of pediatric and adult donors to compare their replicative capacity [34]. They showed no differences in morphology, whereas the cell growth was strictly dependent on the donor´s age, with a twice higher population doubling time in the pediatric population compared to the adult cells. Psaroudis et al. compared the levels of expression of the senescence marker CD26, also known as adenosine deaminase complexing protein 2, in MSCs isolated from the adipose tissue of adult and pediatric donors [35]. This showed that CD26 expression and, accordingly, senescence levels were higher in early passage adult MSCs compared to pediatric MSCs. Moreover, enrichment of CD26 was shown to correlate with impaired immunopotency, i.e., MSC inhibition of proliferating T cells. 

In addition to donor age, health status, and functional deficiencies, basic treatment (with, e.g., corticoids) of patients can also affect the efficacy of autologous or allogeneic MSC treatment. 

MSCs harvested from multiple sclerosis patients showed similar osteogenic and adipogenic differentiation in vitro. This, however, comes with higher senescence, low secretion levels of anti-inflammatory cytokines including interleukin 10 (IL-10) and the transforming growth factor β (TGF-β), modulation of the fibroblast growth factor (FGF) and the hepatocyte growth factor (HGF) signaling pathways. Moreover, they showed decreased inhibition of T cell proliferation compared to healthy individuals [36]. These alterations could not be reversed by autologous hematopoietic stem cell transplantation [36]. Bone marrow-derived cells isolated from patients with myelodysplastic syndrome displayed reduced clonality and growth, elevated senescence, altered osteogenic and adipogenic differentiation potentials, and also abnormal phenotypical characteristics such as higher expression rates of CD29 and CD166 in comparison to healthy MSCs [37]. Adipose-derived MSCs from obese patients showed altered plasticity, manifesting itself in a changed pattern of surface markers both before and after differentiation, including the higher expression of CD106 and HLA-II, the lower expression of CD29, and a decreased cell proliferation and differentiation potential compared to MSCs isolated from lean donors [38]. This might result from the latent effects of the obesity-related hypoxia environment [38]. 

A similar pattern in terms of altered multipotency was observed in experiments with obese mice, thus supporting the hypothesis that this might be regulated by the increased systemic levels of free fatty acids and further obesity-related cytokines [39].

MSCs derived from the bone marrow of osteoporosis patients revealed a similar morphology and surface markers compared to cells isolated from healthy individuals and, at the same time, lower proliferation rates in response to insulin-like growth factor-1 (IGF1) and a deficient osteogenic potential due to an upregulated expression of alkaline phosphatase and calcium phosphate deposition [40]. MSCs derived from osteoporotic donors were characterized by impaired expression and maintenance of collagen type I in the extracellular matrix; there were up to 50% fewer cells compared to healthy donors, combined with higher levels of gelatinolytic activity and decreased expression of TGF-β1, thus leading to a stronger adipogenic differentiation potential [41].

Donor gender-related differences were also reported, i.e., female BM-MSCs were found to have higher population doubling times than male BM-MSCs, with a significant correlation between doubling time and donor age in contrast to cells isolated from male donors [42]. Additionally to differences in the proliferation capacity and cell yields, a study conducted with human MSCs isolated from Wharton´s jelly (WJ-MSCs) showed gender-related differences in the gene expression patterns in terms of a decreased expression of the tumor necrosis factor receptor 1 (TNFR1) and the pro-inflammatory cytokines tumor necrosis factor α (TNFα) and interleukin-1 β (IL-1β) in the female cells [43]. Other groups suggested that female MSCs secrete more anti-inflammatory and pro-angiogenic factors in comparison to male MSCs and thus have a greater therapeutic capacity for vascular remodeling and reducing neonatal hyperoxia-induced lung inflammation [44]. Moreover, female BM-MSCs revealed decreased adipogenic differentiation potential with increasing donor age in comparison to their male counterparts [42]. These findings indicate the necessity of considering donor characteristics, in particular age and gender bias, when selecting MSCs for allogeneic transplantation for meaningful therapeutic outcomes. 

## 3. Tissue Source-Dependent Heterogeneity

MSCs currently used in the field of tissue engineering or other clinical applications can be isolated from different tissues such as the bone marrow, adipose tissue, cord blood, umbilical cord, synovial membrane, lung periosteum, dental pulp, and others (Table 2) [45]. Depending on their source of isolation, MSCs show disparities in their phenotype, proliferation, differentiation capacity, immunomodulatory properties, transcriptional profiles, and proteomic profiles. Unfortunately, biological properties mainly in the skeletal system are based on in vitro assays using cultures that are chemically directed towards osteogenic, chondrogenic, and adipogenic differentiation employing strong induction [9]. Therefore, these tests are not stringent and fail to predict the in vivo differentiation potential of MSCs derived from different tissues. Depending on their tissue source, differentiation of MSCs into osteogenesis, chondrogenesis, or adipogenesis might not even be the correct biological function.

Isolation of MSCs from adult tissues such as the bone marrow encounters several limitations, such as low cell numbers, age- and donor-dependent differences, limited donors, and limitations to autologous use. MSCs isolated from fetal tissue have several advantages over adult MSCs in terms of availability (higher cell numbers and frequency) and cellular proliferation, with lower senescence levels and faster population doubling times [46]. Moreover, their differentiation capacity, though heterogeneous between the different fetal sources [47], is superior compared to adult MSCs, for example, higher basal expression of 16 osteogenic genes in correlation with higher in vitro calcium production [46,48], colony-forming capacity [46], and paracrine effects. 

### 3.1. MSCs from Adult Sources

#### 3.1.1. Bone Marrow-Derived MSCs (BM-MSCs)

With regard to their differentiation potential, BM-MSCs are considered to have a higher tendency to differentiate into osteoblasts [49,50] and into bone and cartilage in vivo [5,51]. This was recently confirmed by Hochmann et al. [52] and can be additionally modulated in vitro by cell culture under hypoxic conditions [53]. In the context of tendinopathy treatment, BM-MSCs appear to be the most suitable source since they show an increased expression of various factors associated with tenogenesis, including collagen I, Scleraxis, and Tenomodulin [54]. 

BM-MSCs are also the most studied cells in the field of cartilage regeneration. The reason for this is the higher chondrogenic potential of cells isolated form the iliac crest and vertebral body in comparison to cells harvested from the femoral head [55]. Hochmann et al. investigated the molecular mechanisms underlying transcriptional stromal differentiation networks and showed that binding sites of commonly expressed transcription factors in the enhancer and promoter regions of ossification-related genes such as Runt and bZIP are only accessible in BM-MSCs and not in other extra-skeletal MSCs, thus suggesting an epigenetically organ-dependent and predetermined differentiation potential [52].

Moreover, BM-MSCs possess the shortest culture periods and the lowest proliferation rates and population doubling time in comparison to cells from other tissues [56], which is enhanced by the in vitro acquired culture-induced aging through gradual telomere shortening and amplified susceptibility to oxidative stress [57]. An additional major disadvantage of BM-MSCs consists of the negative correlation of their differentiation capacity with donor age, which could be inefficient when harvested from elderly patients [32]. 

BM-MSCs were shown to induce anti-fibrotic and anti-inflammatory events after transplantation into the renal sub-capsular area of rats that lead to renal fibrosis reversal and promotion of renal morphological restoration and remodeling, which is achieved by the reduction of collagen deposition, macrophage accumulation, TNF-α reduction, increase of IL-10 expression, Bowman’s capsule, and tubule-interstitial basal membrane morphological recovery [58]. Moreover, they were shown to significantly inhibit allogeneic T cell proliferation through the expression of higher levels of IL-10, TGF-β1 and immunosuppressive cytokines [49]. Other studies also demonstrated the advantages of BM-MSCs in their ability to secrete higher amounts of stem cell-derived factor-1 (SDF-1), which is related to a stronger migration capacity, and HGF, which must be systematically considered during therapeutic applications to increase the efficiency of homing towards the injury site to induce tissue repair [59]. After transplantation in an immunodeficient mice model, BM-MSCs were shown to initiate defect bone healing through secretion of osteopontin, thus contributing to transient mineralized bone hard callus formation [52].

Several clinical trials using allogeneic or autologous BM-MSC injection or transplantation for treatment of various diseases (Table 3). Bolli et al. showed that transendocardial administration of allogeneic BM-MSCs was safe and tolerated by cancer survivors with anthracycline-induced cardiomyopathy, thus providing groundwork for future clinical studies [60]. Moreover, the immunomodulatory effects of BM-MSCs were used for treatment of patients suffering from ischemic injury in numerous clinical trials and have been proved to be beneficiary [61]. Additionally, new treatments have emerged using BM-MSCs for treatment of multiple sclerosis and have also been proved to be efficacious.

#### 3.1.2. Adipose Tissue-Derived MSCs (AT-MSCs)

Adipose tissue is another alternative, less invasive source for the isolation of higher initial yields of MSCs than from the bone marrow, with higher proliferative capacity in vitro [57]. This was first described by Zuk et al. in 2001 [66]. AT-MSCs are isolated from the lipoaspirate obtained during several surgical processes, such as liposuction or lipectomy, which are considered as minimally invasive procedures [67]. They also constitute up to 3% of all cells in the adipose tissue [68,69]. Similar to BM-MSCs, it has been reported that donor age negatively affects the expansion and differentiation potential of AT-MSCs [70]. However, AT-MSCs have also been shown to secrete several factors that support tissue regeneration, such as vascular endothelial cell growth factor (VEGF) and HGF, thus having beneficial effects that can be used for cell-based cardiovascular gene therapy of ischemic tissue [71]. Todorova et al. demonstrated that AT-MSCs are more potent immune modulators of the differentiation of monocyte-derived dendritic cells in comparison to BM-MSCs [72], and others reported AT-MSCs to have a stronger suppressive effect in terms of T cell formation and activation [73]. 

AT-MSCs have emerged as an effective treatment for Crohn´s disease (CD), a condition characterized by chronic inflammation of the gastrointestinal tract with relapsing behavior, no known reasons, and no effective treatments. Allogeneic AT-MSCs have been used for the treatment of complex perianal fistulas in adult patients and are nowadays commercially available in Europe under the name Alofisel^TM^ [74,75,76,77,78]. 

In comparison to BM-MSCs, AT-MSCs exhibit lower chondrogenic and osteogenic potentials [59,79]. However, they show a higher proliferative capacity [57,59,80], a later occurrence of cellular senescence [81], higher immunomodulatory effects, and an upregulated expression of different cytokines, chemokines and growth factors including interferon-γ (IFNγ), basic fibroblast growth factor (bFGF) and IGF-1 [59]. For these reasons, they are nowadays widely used in cartilage regeneration therapies. 

#### 3.1.3. Endometrium-Derived MSCs (E-MSCs)

After its first description by Prianishnikov in 1978 [82], human endometrial tissue has become an interesting MSC source for cell-based therapies due to its easy harvesting techniques without analgesic requirements. Several studies investigated the chondrogenic differentiation potential of E-MSCs for possible application in cartilage regeneration and showed that they could produce abundant amounts of sulfated glycosaminoglycans and type II collagen [83,84,85]. E-MSCs are also characterized for their reduced immunogenic and inflammatory properties in terms of low HLA-ABC and negative HLA-DR expression [86]. Moreover, they could inhibit proliferation, of mouse spleen lymphocytes and human peripheral blood lymphocytes during co-culture due to potential TGF-β1 secretion [86]. A new in vivo study conducted in mice showed that E-MSCs but not AT- or UC-MSCs, could suppress malignant endometrial cancer through inhibition of the Wnt/β-catenin signaling pathway by secreting high levels of Dickkopf-related protein 1 (DKK1) [87]. E-MSCs were also shown to possibly inhibit dendritic cell maturation and proliferation through increased expression of IL-6 and IL-10 [88]. 

These results suggest that E-MSCs have great potential and a promising future for clinical applications. However, only preliminary studies are available, and the lacking mechanisms of action still need to be elucidated.

#### 3.1.4. Synovial Membrane-Derived MSCs (SD-MSCs)

The synovial, membrane or synovium, is the connective tissue that lines the synovial joint cavity. Bari et al. characterized, in 2001, MSCs isolated from the synovial membrane of human knee joints and reported their multi-lineage differentiation potential and in vitro expansion over at least 10 passages with limited cell senescence independently of donor age [89]. SD-MSCs are more accessible and can be extracted during knee surgery or joint aspiration in a minimally invasive procedure for autologous transplantation. Moreover, they have been shown to possess high proliferation rates, reduced immunogenicity through a reduced expression of HLA-DR in comparison to BM-MSCs, and a high chondrogenic potential in comparison with MSCs from other sources [90]. For this, they are studied for possible applications in osteoarthritis therapy by intra-articular injection [91] and in cartilage and meniscus regeneration. The promising potential of SM-MSCs in the treatment of osteoarthritis, which is caused by joint degradation with increasing age and has a higher incidence in females, has increased in the last decade [92]. Several studies reported a reversed osteoarthritis process, improvement of joint motility, cartilage quality, and pain relief [93,94,95,96]. 

#### 3.1.5. Dental Tissue-Derived MSCs (D-MSCs)

MSCs were first isolated from dental pulp but can also be derived from several other adult dental tissues, including exfoliated deciduous teeth, periodontal ligament, apical papilla, gingiva, dental follicle, tooth germ, and alveolar bone. In addition to their ability to control the odontogenic differentiation potential, they are also known for their osteogenic, adipogenic, and chondrogenic differentiation capacities, as well as their transdifferentiation capacities into the ectodermal or endodermal lineages [97].

D-MSCs are increasingly being used in the field of regenerative medicine, with emerging evidence for their better and more impactful immunomodulatory properties. Previous reports showed that D-MSCs can suppress T cell proliferation, which might be suitable for usage during hematopoietic or solid-organ allogeneic transplantation [98]. D-MSCs also inhibited peripheral blood mononuclear cell (PBMNC) proliferation stimulated with mitogen or in an allogeneic mixed lymphocyte reaction (MLR), whereas co-culture with activated PBMNCs led to the upregulation of TGF-β, HGF, and IDO expression after stimulation with IFNγ [99]. The application of D-MSCs in preclinical studies and clinical trials for regenerative therapies for the treatment of dental diseases but also of neurodegenerative [97,100], autoimmune [98,101], and orthopedic [102] disorders is promising. 

### 3.2. MSCs from Fetal Sources

#### 3.2.1. Cord Blood MSCs (CB-MSCs)

Rubinstein et al. first reported in 1993 the use of frozen stored placental blood as an alternative source for hematopoietic stem cells for unrelated bone marrow reconstitution [103]. Within the last decades, placental cord blood has been widely established as a valuable source for both hematopoietic stem cells and mesenchymal stromal cells. Different groups did not succeed in isolating MSCs from cord blood in contrast to the bone marrow [104,105]. Others described methods for the successful isolation of MSCs from umbilical cord blood despite low cell frequency [106,107] and that could even reach in vitro differentiation into different lineages [108]. MSCs isolated from cord blood have been shown to have a unique chondrogenic differentiation potential in vivo and reveal higher replicative rates compared to BM-MSCs [51,57,109].

Several studies showed that CB-MSCs, compared to BM-MSCs and AT-MSCS, have a reduced adipogenic differentiation potential, which might be related to the vast amounts of pre-adipocyte factor 1 (Pref-1) in cord blood plasma, which confers CB-MSCs anti-adipogenic properties [110,111]. This can, however, be adjusted by negative regulation of the Wnt5a/β-catenin signaling pathway through exogenous calcium treatment [68].

In addition to CB-MSCs, cord blood also contains a population of previously named “unrestricted somatic stem cells” (USSC), which are characterized by the absence or marginal expression of all 39 *HOX*-genes in contrast to CB- or BM-MSCs [112,113]. In humans, the 39 *HOX* genes are located in four different clusters: A, B, C, and D, as first described by Krumlauf in 1994 [114]. While regulated HOX expression is important during embryonic and fetal development [115], Ackema and Charite described the HOX code for MSCs derived from different anatomic sites [116]. Our group was able to show that BM- and CB-MSCs expressed the HOX code in all four clusters, unlike USSCs [112]. This reflects the fact that the USSCs originate from a different biological niche during fetal development. Moreover, our group demonstrated that the expression levels of the δ-like 1/pre-adipocyte factor 1 (DLK-1/PREF1) also allows the distinction between USSCs and CB-MSCs [117]. Accordingly, when DLK-1/PREF1 was constitutively expressed in CB-MSCs, the adipogenic differentiation potential was impaired, whereas its silencing in USSCs allowed adipogenesis [117]. Subsequently, CB-MSCs and USSCs derived from cord blood must be clearly distinguished from umbilical cord-derived MSCs, since UC-MSCs fail to differentiate in vitro and in vivo towards bone and cartilage and also differ in their respective HOX expression patterns [118]. 

#### 3.2.2. Umbilical Cord-Derived MSCs (UC-MSCs)

Similar to CB-MSCs, UC-MSCs can also be extracted without any ethical controversies from umbilical cord tissue after childbirth and display a four time higher proliferation levels compared to BM-MSCs and AT-MSCs [119]. In an attempt to characterize UC-MSCs, UC-derived primary cells with mesenchymal-like properties separated by counterflow centrifugal elutriation displayed several subpopulations differing in their sizes and proliferation potentials. These may be precursors of the mature populations or are probably connected to the amount of senescent cells in the respective populations [120].

Although UC-MSCs have different molecular chondrogenic and osteogenic signatures lacking substantial integrin-binding sialoprotein expression [121] and skeletal formation in vivo [118], UC-MSCs have been extensively used in clinical research related to neurodegenerative and cerebrovascular diseases, autism, spinal cord injury, and hypoxic ischemic encephalopathy (Table 4). 

Intracerebral transplantation of UC-MSCs was shown to alleviate encephalopathy caused by neonatal hypoxia and ischemia in rat neonates by in vitro inhibition of apoptosis of injured neurons [122]. In hyperoxia-exposed rats, UC-MSCs lead to a greater improvement of alveolarization and less macrophage infiltration compared to BM-MSCs [123]. 

Min et al. determined that UC-MSCs can be potentially used for therapy of demyelinating diseases of the central nervous system since they could promote spinal cord re-myelination by suppressing neuro-inflammation through interaction with microphages and suppressing microglial cell interaction, resulting in a reprogramming of the immune response in a mouse model [124]. The exact molecular mechanism responsible for this interaction is, however, not yet resolved [124,125]. Other groups described the application possibilities of the re-myelination properties of UC-MSCs for the treatment of multiple sclerosis [125,126]. Wehbe et al. reported the usage of allogeneic UC-MSCs for the treatment of progressive and refractory scleroderma, with a combined immunotherapy approach resulting in a significant overall improvement [127].

**Table 4:** Examples of clinical trials of UC-MSCs for treatment of various conditions.

Condition	MSC	Phase	Clinical Trial	Status	References
Intraventricular Hemorrhage	Allogeneic UC-MSC (intraventricular injection)	Phase I	NCT02274428	Completed	[128]
Bronchopulmonary Dysplasia (BPD)	Allogeneic UC-MSC (intratracheal injection)	Phase I	NCT01632475	Active, not re-cruiting	[129]
Cerebral Palsy	Allogeneic CB- and UC-MSC	Phase I/II	NCT03473301	Completed	-
Hypoxic-Ischemic Encephalopathy	Allogeneic UC-MSC	Pilot phase I	NCT03635450	Completed	[130]
Bronchopulmonary dysplasia (BPD)	UC-MSC	Phase II	NCT01828957	Completed	[131]
Myocardial Infarction	Allogeneic UC-MSC	Phase I	NCT03798353	Completed	-
Autism	UC-MSC	Phase II	NCT04089579	Active, not recruiting	-
	Allogeneic UC-MSC	Phase I	NCT03099239	Completed	[132]

#### 3.2.3. Placenta-Derived MSCs (P-MSCs)

The placenta is a feto-maternal organ that is usually discarded post-partum, thus its easy availability and non-invasive harvesting. Recently, it has been shown that several parts of the placenta are rich and sustainable MSC sources unlike the bone marrow [133]. In comparison to BM-MSCs, P-MSCs showed a higher replicative capacity and broader differentiation abilities, which are related to the placental function of supporting fetus growth [134].

MSCs derived from the fetal tissues of the placenta have been used in animal disease models of several disorders such as cancer, liver diseases, cardiac disorders, ulcers, bone diseases, neurological diseases, and more recently, coronavirus (COVID-19). They are widely available and characterized by a high secretion of paracrine effects, a low immunogenicity, and low risk of senescence. However, the molecular mechanisms of their specific immunomodulatory properties are still not elucidated.

#### 3.2.4. Amniotic Fluid-Derived MSCs (AF-MSCs)

The amniotic fluid is a rich source of fetal cells, including MSCs. It can be collected either invasively during pregnancy by amniocentesis from second trimester amniotic fluid, which might result in fetus infection, or during a C-section. These populations might, however, differ in terms of potency, maturity, and plasticity since they originate from two different pregnancy timepoints [135]. AF-MSCs harvested during C-section were characterized by Spitzhorn et al [135]. They were shown to meet the MSC criteria described by the ISCT. AF-MSCs were shown in various studies to express the pluripotency factor Oct4, but this could not be confirmed by Spitzhorn et al. [135,136,137]. Moreover, these findings remain controversial since the self-renewal function of Oct4 has not yet been defined in AF-MSCs, and the studies rather focus on the expression without addressing the function of Oct4 [135,138]. AF-MSCs were also shown to express the early embryonic glycolipid antigens SSEA4 and c-Kit, which are necessary for the maintenance and differentiation of the hematopoietic stem cells [135]. Analysis of AF-MSC-conditioned media revealed the presence of several pro- and anti-angiogenic factors, i.e., vascular endothelial growth factor (VEGF), interleukin 8 (IL-8), and IFNγ [139]. Moreover, Mirabella et al. showed that AF-MSCs do not directly contribute to bone formation but do contribute to the vascular modeling of the engineered bone [139]. The underlying mechanisms are still not elucidated.

## 4. Culture Conditions-Dependent Heterogeneity

In addition, donor variations and differences in the sources of isolation, MSC heterogeneity is also strongly dependent on their culture conditions. Hereby, several factors must be considered, including culture medium, O_2_ tension, mechanical stimuli, inflammatory stimuli, and mechanical cues [140]. 

### 4.1. Culture Medium

MSCs cultured in vitro can undergo morphological, phenotypical, and genetic changes with increasing passage numbers. This can be additionally modulated by the composition of the culture medium, which was shown to influence senescence levels and differentiation capacity [141]. Nowadays, several culture media and technologies are used for the expansion of MSCs, such as fetal bovine serum (FBS) or xeno-free or chemically defined media, to avoid FBS batch-to-batch differences.

### 4.2. O_2_ Tension

MSCs are generally cultured in vitro under normoxic conditions, despite the fact that biological niches such as the adipose or the bone marrow niches are adapted to hypoxic O_2_ tensions. Our group and several others showed O_2_ tension-dependent differences in the proliferative capacities, surface marker expression profiles, and differentiation capacities of different types of MSCs [142,143,144,145]. In addition to this, the hypoxic environment offers protection against replicative senescence and damaging factors [146]. It has also been reported that low oxygen levels can also facilitate the release of trophic factors and angiogenesis growth factors, thus contributing to the improvement of ischemic injuries [140,147,148]. 

## 5. Human Induced Pluripotent Stem Cell (iPSC)-Derived MSCs (iMSCs)

A robust expansion of therapeutic numbers of MSCs is frequently hard to achieve in an autologous setting due to higher senescence, DNA damage accumulation, genome instability, and oxidative stress. These factors challenge the manufacturing possibilities of homogenous and large numbers of MSC products, both for research and for the development of cell-based therapies. iPSCs derived from MSCs have been proposed as a clinically relevant alternative to bypass these limitations by suppressing the existing mechanical memory, which stores epigenetic and transcriptional information from the past environment that biases the cell fate [149]. Additionally, they are theoretically unlimited in supply and are more convenient for genetic modulation, scale-up production, and quality control. iPSCs with different tissue and reprogramming backgrounds could be differentiated into different types of somatic cells, including mesenchymal progenitors that have similar properties to somatic tissue-derived MSCs [150]. In the last decade, human iMSCs have been successfully used for improvement of bone regeneration in mice and mini-pigs [151,152,153], promotion of mucosal healing in mouse models of inflammatory bowel disease [154], and treatment of skin ischemia in mouse models [155].

A simple one-step protocol for the generation of MSCs from iPSCs exhibiting MSC characteristics, including expression of surface markers and trilineage differentiation potential, has been suggested by Zhou et al [156]. These results support the potential application for industrial-scale production of iMSCs. Zhou et al. showed that iMSCs were similar in their morphology, immune phenotype, in vitro differentiation potential, DNA methylation patterns, prevention of bone loss, and promotion of bone repair to BM-MSCs [73]. However, their tumorigenic capacity increased, although their proliferation rate was higher. Furthermore, their transplantation into rats with osteonecrosis of the femoral head effectively led to the promotion of bone repair and the prevention of bone loss. Eto et al. showed that MSCs derived from iPSCs could suppress cartilage degeneration and improve joint destruction in an osteoarthritis model [155]. Ozay et al. reported that administration of iMSCs in a humanized mouse model of GvHD led to reduced disease severity and prolonged survival [157]. The mechanisms of action are, however, not yet clearly elucidated. Bloor et al. conducted a Phase I trial using iPSC-derived MSCs (NCT02923375) in subjects with steroid-resistant acute GvHD to investigate their safety and tolerability [158]. They were shown to be safe and well tolerated by all patients, which is a great advantage for possible applications in diverse other inflammatory diseases. 

## 6. Conclusions and Future Perspectives

This review summarizes two of the factors that mainly affect MSC heterogeneity, namely donor and tissue source, thus constituting a limiting factor inhibiting the exploitation of their full potential in therapeutical applications and industrialization. Growing evidence emerged in the last decade supporting the immunomodulatory features of MSCs, and various clinical trials with different experimental settings showed that administration of MSCs is in fact beneficial. For this, further research needs to be developed to establish new methods to eliminate or control this inherent heterogeneity and standardize MSC production for clinical applications. For clinical application, MSC potency needs to be determined and is defined by the therapeutical activity of a cell/cell population as indicated by appropriate laboratory tests or adequately developed and controlled clinical data. This potency is independent from the classical criteria of MSC to form bone, cartilage, and adipose tissue but is instead based on paracrine effects, cytokine release, surface and homing markers, as well as various other mechanisms as documented and granted by the US FDA for the treatment of neurological conditions in children. In contrast to paracrine mechanisms for neurological disorders, bone and cartilage formation requires a distinct cellular repertoire and signature for regeneration in vivo, as described by our group [51] and Hochmann et al. [52].

## Figures and Tables

**Table 1:** Positive and negative markers of MSCs.

**Positive Markers**	**Physiological Function**
CD44	Hyaluronic receptor, surface adhesion, migration
CD73	Lymphocyte-vascular adhesion protein 2 (Ecto-5’-nucleotidase)
CD90	Cell adhesion, migration, apoptosis, fibrosis, T cell activation
CD105	Activation and proliferation of endothelial cells
CD106	Vascular cell adhesion molecule-1 (VCAM-1)
CD146	Melanoma cell adhesion molecule (MCAM)
**Negative Markers**	**Physiological Function**
CD11b	Integrin αM subunit, NK Cells, neutrophils, monocytes, macrophages
CD14	Lipopolysaccharide receptor, macrophages, monocytes
CD19	B cell lymphocytes
CD34	Adhesion molecule, hematopoietic stem cell
CD45	B cell lymphocyte receptor complex
CD79	B cell lymphocyte and B cell neoplasms
HLA-DR	MHC class II cell surface receptor

**Table 2:** List of the main sources for isolation of MSCs, the respective isolation technique, and culture conditions. Yellow: adult tissues. Red: fetal/perinatal tissues.

MSC Source	Isolation Technique
Bone marrow	Density gradient centrifugation, Ficoll gradient or red blood cell lysis of bone marrow aspirate
Adipose tissue	Enzymatic or non-enzymatic digestion after liposuction or lipectomy
Endometrium	Enzymatic digestion after scraping the myometrium of hysterectomy samples
Synovial membrane	Enzymatic digestion of synovium harvested from the inner joint side
Dental tissue	Extirpation of dental pulps after decoronation
Cord blood	Direct expansion
Umbilical cord	Enzymatic digestion or direct expansion of umbilical cord tissue
Wharton’s jelly	Vein removal, scraping and enzymatic digestion
Placenta	Enzymatic digestion
Amniotic fluid	Amniotic membrane perforation and tubing for fluid collection followed by density gradient centrifugation

**Table 3:** Examples of clinical trials of BM-MSCs for treatment of various conditions.

Condition	MSC	Phase	Clinical Trial	Status	References
Multiple Sclerosis	Autologous BM-MSC	Phase II	NCT02166021	Completed	[62]
	Autologous BM-MSC	Phase II	NCT02239393	Completed	[63,64]
Post-traumatic Pulp Necrosis	Allogeneic BM-MSC	Phase II/III	NCT04545307	Completed	-
Anthracycline-induced cardiomyopathy	Allogeneic BM-MSC	Phase I	NCT02509156	Completed	[60]
SR-aGvHD	BM-MSC	Phase III	NCT02336230	Completed	[65]
Liver Cirrhosis	Autologous BM-MSC	Phase III	NCT05080465	Completed	-
Covid-19 Infection	Allogeneic BM-MSC	Phase I	NCT04397796	Active, not recruiting	-
Chronic Myocardial Ischemia	Autologous BM-MSC	Phase II	NCT02462330	Completed	-

## Data Availability

Not applicable.
